# External validation of the Prehospital Return of Spontaneous Circulation (P-ROSC) score for predicting prehospital return of spontaneous circulation among patients with out-of-hospital cardiac arrest

**DOI:** 10.1016/j.resplu.2026.101294

**Published:** 2026-03-19

**Authors:** Ryuta Onodera, Norihiro Nishioka, Yohei Okada, Yuto Makino, Kosuke Kiyohara, Tetsuhisa Kitamura, Taku Iwami

**Affiliations:** a Department of Preventive Services, Kyoto University School of Public Health, Kyoto, Japan; b Department of Nephrology and Rheumatology, Kyorin University School of Medicine, Tokyo, Japan; c Prehospital and Emergency Research Centre, Health Services Research and Population Health, Duke-NUS Medical School, Singapore; d Department of Anesthesiology and Intensive Care Medicine, Nagoya City University Graduate School of Medical Sciences, Nagoya, Japan; e Department of Food Science, Otsuma Women’s University, Tokyo, Japan; f Division of Environmental Medicine and Population Sciences, Department of Social and Environmental Medicine, Graduate School of Medicine, Osaka University, Osaka, Japan

**Keywords:** Out-of-hospital cardiac arrest, Prehospital return of spontaneous circulation, Emergency medical services, Prediction model

## Abstract

**Background:**

The Prehospital Return of Spontaneous Circulation (P-ROSC) score was developed using the Pan-Asian Resuscitation Outcomes Study registry to predict prehospital ROSC. Although external validation studies have been conducted in other Asian countries, their results have been inconsistent. This study aimed to externally validate the P-ROSC score using a nationwide Japanese registry and assess its performance across geographic regions.

**Methods:**

We analysed out-of-hospital cardiac arrest (OHCA) cases of medical origin registered in the All-Japan Utstein Registry between 2015 and 2020. The primary outcome was prehospital ROSC, defined as ROSC at the scene or during transport. Model discrimination was evaluated using the area under the receiver operating characteristic curve (AUC), and calibration was assessed using calibration plots. Subgroup analyses were conducted across eight regions of Japan.

**Results:**

A total of 493,695 OHCA cases were included, with a median age of 81 years, and 56.5% were men. Overall, 46,760 patients (9.5%) achieved prehospital ROSC. The AUC of the P-ROSC score based on the predefined model was 0.794 (95% confidence interval [CI]: 0.792–0.796). The calibration plot showed moderate agreement between predicted and observed outcomes. The region-specific AUCs varied across Japan, with the highest AUC in Tohoku (0.822, 95% CI: 0.816–0.828) and the lowest in Hokkaido (0.772, 95% CI: 0.764–0.780).

**Conclusions:**

The P-ROSC score demonstrated fair discrimination and moderate calibration for predicting prehospital ROSC in Japanese patients with OHCA. Its application should consider regional variations in patient characteristics and emergency medical service structures that may influence transportation and generalisability.

## Highlights

• We validated the P-ROSC score using a nationwide Japanese OHCA registry.
• The P-ROSC score showed acceptable discriminative ability and moderate calibration for predicting prehospital ROSC.
• Its predictive performance varied across regions, supporting its use with attention to regional heterogeneity.

## Introduction

Out-of-hospital cardiac arrest (OHCA) remains a major public health concern worldwide, with low survival rates despite continuous improvements in emergency medical service (EMS) systems.^1^ Accurate prognostic assessment in the prehospital setting is essential for guiding clinical decision-making, optimising triage, and facilitating effective communication with receiving hospitals. Several prediction models have been developed to estimate the likelihood of prehospital return of spontaneous circulation (ROSC).^2^, ^3^, ^4^, ^5^ Among these, the ROSC after Cardiac Arrest (RACA) and Utstein-Based ROSC (UB-ROSC) scores are widely recognised. However, both methods require numerous variables and complex calculations, which limit their immediate applicability in real-world emergency settings. Moreover, these models incorporate factors such as the aetiology of cardiac arrest, which is often difficult to ascertain in a prehospital environment.

To address these challenges, a prehospital ROSC (P-ROSC) score was developed using data from the Pan-Asian Resuscitation Outcomes Study (PAROS) registry.^6^, ^7^ The derivation cohort included patients from multiple Asian regions, including major urban areas in Japan. This score was constructed using AutoScore—a machine learning–based framework—and consists of only five clearly defined variables: age, witnessed status, response time, initial rhythm, and prehospital drug administration. In addition to its simplicity, the P-ROSC score demonstrated a high discriminative ability in the development cohort (area under the receiver operating characteristic curve [AUC]: 0.806; 95% confidence interval [CI]: 0.799–0.814), outperforming the RACA and UB-ROSC scores.^6^

Although external validations of the P-ROSC score have been reported, the existing evidence remains limited and inconsistent. Prior validation studies conducted in Taiwan and Thailand yielded heterogeneous results, with reported AUC values of approximately 0.76 in Taiwan and 0.64 in Thailand.^8^, ^9^ These differences may reflect variations in baseline outcome prevalence, case-mix, and prehospital care processes across the study populations. Moreover, these studies were based on relatively small or selected cohorts, which may restrict the generalisability of their findings. Consequently, the robustness and transportability of the P-ROSC score across broader, population-based settings remain uncertain.

Notably, the variables included in the P-ROSC score reflect patient-related characteristics, such as age and initial rhythm, and system- and process-related factors, including EMS response time and prehospital drug administration. These system-dependent variables may be influenced by local EMS organisation, resource availability, and treatment protocols. The All-Japan Utstein Registry is one of the largest nationwide population-based OHCA registries. Although the Japanese EMS system is nationally standardised, regional differences exist in EMS response times, allocation of medical resources, and implementation of prehospital interventions.^10^, ^11^, ^12^, ^13^ Consequently, regional heterogeneity may influence the predictive performance of the P-ROSC score. Moreover, given that the derivation of the P-ROSC score incorporated data from only selected major urban areas in Japan, its performance within a single national EMS system cannot be assumed to be uniform.

Therefore, this study aimed to externally validate the P-ROSC score using a nationwide Japanese registry and explore regional differences in its predictive accuracy. This validation study provides valuable insights into the generalisability and real-world utility of the P-ROSC score in guiding prehospital resuscitation strategies.

## Methods

### Study design

This study was designed as a geographically independent external validation of the P-ROSC score using a nationwide Japanese registry and is reported in accordance with the Transparent Reporting of a multivariable prediction model for Individual Prognosis or Diagnosis (TRIPOD) statement (Table S1).^14^ The study protocol was approved by the Ethics Committee of Kyoto University (approval no. R1538-2). Personal identifiers were removed from the Fire and Disaster Management Agency (FDMA) databases. Therefore, the requirement for informed consent from patients was waived.

### Settings

Previous reports have described the All-Japan Utstein Registry of the FDMA in detail.^15^ This prospective, population-based registry includes patients with OHCA and follows the international Utstein style.^16^, ^17^, ^18^ Additional information on the All-Japan Utstein Registry is provided in Supplementary Appendix 1.

### Participants

This validation study included adult patients aged 18 years or older who experienced OHCA of medical origin, received resuscitation from EMS personnel, and were subsequently transported to medical institutions. The original development of the P-ROSC score incorporated Japanese data collected from major urban areas—Tokyo, Osaka, and Aichi Prefectures—between 2009 and 2014 as part of the PAROS registry. Data were obtained from the All-Japan Utstein Registry from January 2015 to December 2020. The exclusion criteria included cases with an unknown initial electrocardiographic rhythm and cases from Tokyo, Osaka, and Aichi Prefectures, which were included in the original model development study. Patients with OHCA of an external origin—including those with poisoning, drowning, traffic trauma, or hypothermia—were also excluded.

### Outcome measurements

The primary outcome of this study was prehospital ROSC, defined as any palpable spontaneous pulse detected before hospital arrival.^18^ The presence or absence of ROSC was assessed and documented by EMS personnel during field resuscitation or transport.

### Prediction model of interest

In this validation study, the P-ROSC score was applied exactly as described in the original developmental study. The score was calculated using five predictors: age, EMS response time, initial cardiac rhythm, witnessed status, and prehospital drug administration, with higher total scores (range, 0–100) indicating a greater likelihood of prehospital ROSC. Detailed scoring rules and variable definitions are provided in Tables S2 and S3.

### Sample size estimation

To ensure a precise estimate of the discriminative performance of the model during external validation, we calculated the required sample size based on the standard error of the C-statistic, following the method described by Riley et al.^19^ We used an anticipated C-statistic (*I*) of 0.8, an assumed outcome event proportion (*Φ*) of 0.1, and a target standard error of 0.0255, corresponding to a 95% CI width of approximately 0.1. Based on these inputs, the minimum required sample size was calculated to be 837.

### Statistical analysis

We extracted variables included in the P-ROSC score: age, witnessed arrest, initial rhythm at the scene, time from emergency call to EMS arrival, prehospital administration of adrenaline, and achievement of prehospital ROSC. The P-ROSC score was calculated according to the predefined scoring components and point allocations (Table S2).^6^ Patient characteristics were summarised using medians and interquartile ranges (IQRs) for continuous variables and frequencies with percentages for categorical variables.

To assess the discriminatory performance of the P-ROSC score, we calculated the C-statistic (AUC) and its 95% CI. For calibration assessment, we constructed a calibration plot by dividing the predicted probabilities into 15 equally sized groups and compared the predicted and observed outcomes within each group. To further assess the predictive performance of the P-ROSC score, we calculated diagnostic accuracy metrics at every 10-point increment of the P-ROSC score (i.e., ≥10, ≥20, ≥30, and so on). For each cutoff value, we calculated the proportion of patients achieving ROSC, sensitivity, specificity, positive predictive value (PPV), and negative predictive value (NPV), along with their corresponding 95% CIs. To evaluate the clinical utility of the P-ROSC score, decision curve analysis was performed using predicted probabilities derived from a logistic regression model. Rule-in and rule-out strategies were assessed across a range of threshold probabilities. Additionally, subgroup analyses were performed to assess the discrimination and calibration of the P-ROSC score according to sex (male vs. female), bystander cardiopulmonary resuscitation (CPR) (with vs. without bystander CPR), and aetiology of cardiac arrest (cardiac vs. non-cardiac). Because the proportion of missing data was minimal (Table S4), we performed a complete case analysis. To explore regional variability, a subgroup analysis was conducted across eight geographical regions of Japan: Hokkaido, Tohoku, Kanto, Chubu, Kinki, Chugoku, Shikoku, and Kyushu (Fig. S1). Regions were defined based on the prefecture in which the cardiac arrest occurred. This classification is based on commonly accepted regional divisions in Japan that reflect administrative boundaries as well as geographic and cultural distinctions. These regional groupings are widely used in governmental statistics and healthcare policies.^13^, ^20^, ^21^ All statistical analyses were performed using the R software (R Foundation for Statistical Computing, version 4.2.1) and Stata software (version 17; StataCorp, College Station, TX, USA).

## Results

### Patient characteristics

**Fig. 1:**
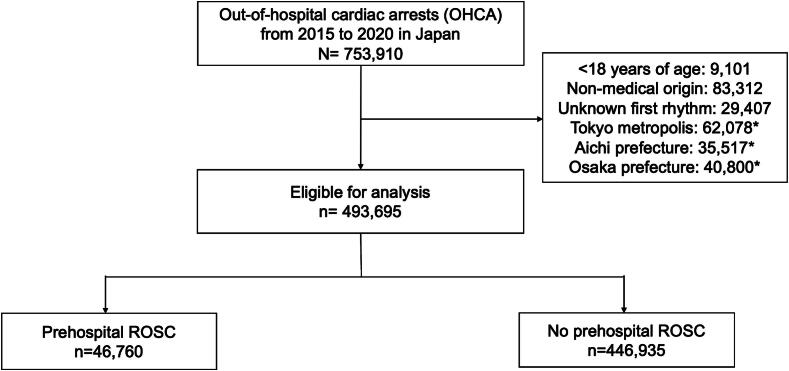
**Study flowchart**. P-ROSC, prehospital return of spontaneous circulation. *These prefectures were excluded because they were included in the original development cohort of the P-ROSC score.

Between 1 January 2015 and 31 December 2020, a total of 753,910 patients with OHCA were recorded in the All-Japan Utstein Registry. Among them, 9101 (1.2%) were younger than 18 years, 83,312 (11.1%) had a non-medical origin, 29,407 (3.9%) had an unknown initial rhythm, and 138,395 (18.4%) were OHCA patients from Tokyo, Aichi, and Osaka Prefectures. In total, 493,695 patients with OHCA were included in the analysis (Fig. 1). The baseline characteristics of patients with OHCA are presented in Table 1. The median age of the patients was 81 years (IQR: 70–88 years), and 56.5% were men. The initial documented rhythm was shockable in 8.5% of cases. Adrenaline was administered by EMS in 25.9% of patients. The median time from emergency call to EMS arrival was 8.0 min (IQR: 6.0–10.0 min). ROSC was achieved in 9.5% of patients, 5.4% survived at 1 month, and 2.8% had favourable neurological outcomes at 1 month.

**Table 1:** Characteristics of patients with out-of-hospital cardiac arrest by region.

**Variables**	**Overall**	**Hokkaido**	**Tohoku**	**Kanto (except Tokyo)**	**Chubu (except Aichi)**	**Kinki (except Osaka)**	**Chugoku**	**Shikoku**	**Kyushu**
*n*	493,695	28,109	55,182	148,598	76,372	66,806	34,075	19,569	64,984
Age	81 [70–88]	80 [69–88]	82 [72–88]	80 [70–87]	82 [71–88]	81 [71–88]	81 [71–88]	81 [71–88]	81 [69–88]
Sex, Male	279,070 (56.5)	15,606 (55.5)	30,673 (55.6)	85,128 (57.3)	43,717 (57.2)	37,766 (56.5)	19,039 (55.9)	10,995 (56.2)	36,146 (55.6)
**Arrest witnessed**									
Lay person	170,300 (34.5)	9050 (32.2)	19,182 (34.8)	50,659 (34.1)	26,548 (34.8)	23,984 (35.9)	11,643 (34.2)	6418 (32.8)	22,816 (35.1)
No	292,653 (59.3)	17,626 (62.7)	32,768 (59.4)	88,711 (59.7)	45,250 (59.2)	38,505 (57.6)	20,096 (59.0)	11,842 (60.5)	37,855 (58.3)
Professional	30,742 (6.2)	1433 (5.1)	3232 (5.9)	9228 (6.2)	4574 (6.0)	4317 (6.5)	2336 (6.9)	1309 (6.7)	4313 (6.6)
Bystander CPR	262,231 (53.1)	13,832 (49.2)	30,799 (55.8)	73,881 (49.7)	41,642 (54.5)	35,962 (53.8)	18,258 (53.6)	9317 (47.6)	38,540 (59.3)
Cardiac cause	343,810 (69.6)	19,866 (70.7)	41,196 (74.7)	111,864 (75.3)	49,065 (64.2)	46,652 (69.8)	22,375 (65.7)	13,367 (68.3)	39,425 (60.7)
**First rhythm**									
Shockable	42,174 (8.5)	2623 (9.3)	4556 (8.3)	12,968 (8.7)	6193 (8.1)	5661 (8.5)	2739 (8.0)	1468 (7.5)	5966 (9.2)
Unshockable	451,521 (91.5)	25,486 (90.7)	50,626 (91.7)	135,630 (91.3)	70,179 (91.9)	61,145 (91.5)	31,336 (92.0)	18,101 (92.5)	59,018 (90.8)
Adrenaline administration by EMS	127,875 (25.9)	10,204 (36.3)	10,895 (19.7)	46,011 (31.0)	20,919 (27.4)	16,911 (25.3)	8208 (24.1)	2594 (13.3)	12,133 (18.7)
Advanced airway management by EMS	209,791 (51.0)	18,918 (79.9)	16,368 (38.6)	70,642 (57.0)	34,314 (51.6)	32,713 (61.0)	13,314 (48.3)	5919 (36.9)	17,603 (30.6)
Time to EMS arrival (minutes)	8.0 [6.0–10.0]	7.0 [6.0–9.0]	8.0 [6.0–11.0]	7.0 [6.0–9.0]	8.0 [6.0–10.0]	7.0 [6.0–9.0]	8.0 [6.0–10.0]	8.0 [6.0–10.0]	8.0 [6.0–10.0]
Time to hospital arrival (minutes)	32.0 [26.0–40.0]	32.0 [26.0–40.0]	33.0 [27.0–42.0]	33.0 [27.0–40.0]	32.0 [26.0–40.0]	31.0 [25.0–38.0]	32.0 [26.0–41.0]	31.0 [25.0–39.0]	30.0 [24.0–38.0]
Prehospital ROSC	46,760 (9.5)	3002 (10.7)	4350 (7.9)	14,976 (10.1)	6920 (9.1)	6739 (10.1)	2889 (8.5)	1235 (6.3)	6649 (10.2)
Survival at 1 month	26,725 (5.4)	1737 (6.2)	2231 (4.0)	7916 (5.3)	3538 (4.6)	4133 (6.2)	1781 (5.2)	930 (4.8)	4459 (6.9)
Favourable neurological survival at 1 month*	14,034 (2.8)	789 (2.8)	1200 (2.2)	4131 (2.8)	2052 (2.7)	1987 (3.0)	887 (2.6)	524 (2.7)	2464 (3.8)

Categorical variables are presented as number (%), and continuous variables as median [interquartile range]. CPR, cardiopulmonary resuscitation; EMS, emergency medical services; ROSC, return of spontaneous circulation.

* Favourable neurological outcome was defined as a Cerebral Performance Category score of 1 or 2.

Baseline characteristics were compared between the development and validation cohorts (Table S5). Compared with the development cohort, patients in the validation cohort were older and more frequently received prehospital drug administration. The median EMS response time was longer in the validation cohort, and the proportion of patients achieving ROSC was slightly higher than in the development cohort.

Table 1 also summarises the baseline characteristics of patients with OHCA across various regions of Japan. Substantial regional variations were observed. For instance, the proportion of patients who received adrenaline via the EMS ranged from 13.3% in Shikoku to 36.3% in Hokkaido. Similarly, the use of AAM varied between 30.6% in Kyushu and 79.9% in Hokkaido. The proportion of patients who achieved ROSC ranged from 6.3% in Shikoku to 10.7% in Hokkaido. One-month survival rates ranged from 4.0% in Tohoku to 6.9% in Kyushu, while the proportion of patients with favourable neurological outcomes at 1 month varied from 2.2% to 3.8% across regions.

### Model performance

**Fig. 2:**
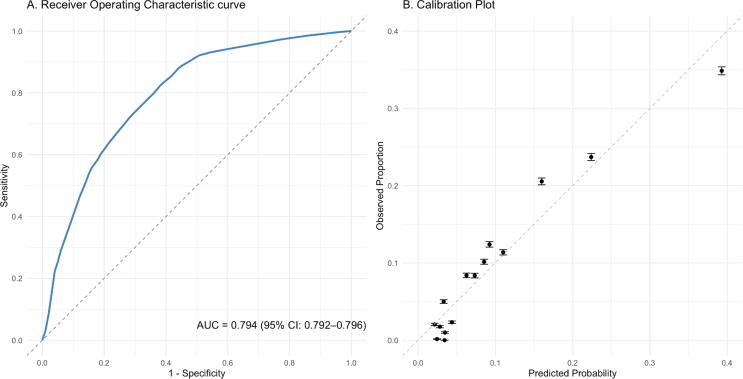
**Discrimination and calibration performance of the P-ROSC score for predicting prehospital ROSC (A) Receiver operating characteristic (ROC) curve for the P-ROSC score. (B) Calibration plot comparing the predicted probabilities of spontaneous circulation with the observed outcomes across deciles. The dashed line represents a perfect calibration. Points and vertical bars indicate observed proportions and 95% confidence intervals, respectively**. P-ROSC, prehospital return of spontaneous circulation.

The predictive performance of the P-ROSC score for prehospital ROSC was evaluated using the receiver operating characteristic curve analysis and calibration plots for the overall cohort and each of the eight geographical regions. The overall AUC was 0.794 (95% CI: 0.792–0.796) (Fig. 2A). Region-specific AUCs were as follows: Tohoku, AUC 0.822 (95% CI 0.816–0.828); Shikoku, AUC 0.813 (95% CI 0.801–0.825); Chubu, AUC 0.806 (95% CI 0.801–0.811); Chugoku, AUC 0.795 (95% CI 0.787–0.802); Kinki, AUC 0.791 (95% CI 0.786–0.796); Kanto, AUC 0.783 (95% CI 0.779–0.787); Kyushu, AUC 0.783 (95% CI 0.777–0.789); and Hokkaido, AUC 0.772 (95% CI 0.764–0.780). These results indicated consistent discriminative performance across regions, with only modest variation (Fig. S2). Calibration plots demonstrated that predicted probabilities reasonably aligned well with the observed proportions across most regions (Fig. S3).

The calibration plot for the overall cohort showed minimal discrepancies between P-ROSC–predicted probabilities and observed outcomes of prehospital ROSC, indicating an overall moderate calibration of the model (Fig. 2B). Predictions were slightly overestimated in the lowest probability group (<0.05), closely aligned with observations in the 0.05–0.25 range, and slightly overestimated again in the higher probability group (≥0.30). Regional calibration plots indicated variability in model calibration across eight regions (Fig. S3).

Evaluation of the prognostic performance at every 10-point increment of the P-ROSC score demonstrated a stepwise trade-off between sensitivity and specificity (Table S6). At a cutoff of ≥10, sensitivity was very high (98.5%, 95% CI: 98.4–98.6), whereas specificity remained low (14.6%, 95% CI: 14.5–14.7). As the cutoff value increased, sensitivity decreased, while specificity improved. The PPV increased with higher score thresholds (from 10.8% at ≥10 to 34.5% at ≥60), while the NPV remained consistently high across all groups, exceeding 90%.

Decision curve analysis demonstrated that the P-ROSC score provided a higher net benefit than treat-all or treat-none strategies across intermediate threshold probabilities. For rule-in decisions, the net benefit was greatest at low to moderate high-risk thresholds (approximately 5–30%). For rule-out decisions, the P-ROSC score showed a higher net benefit than treat-none strategies at low-risk thresholds above approximately 10% (Fig. S4). In subgroup analyses, the P-ROSC score showed consistent discrimination across sex, bystander CPR status, and aetiology of cardiac arrest. The AUCs ranged from 0.789 to 0.795 by sex (Fig. S5), from 0.768 to 0.814 by bystander CPR status (Fig. S6), and from 0.751 to 0.817 by aetiology (Fig. S7). Discrimination was higher among patients with bystander CPR and those with cardiac aetiology. Calibration plots demonstrated an overall good agreement between predicted and observed risks across all subgroups, although slight deviations were observed in higher predicted risk strata.

## Discussion

In this large-scale nationwide external validation study, we evaluated the performance of the P-ROSC score in predicting prehospital ROSC among patients with OHCA in Japan. The P-ROSC score demonstrated fair discriminative performance, with an overall AUC of 0.794 (Fig. 2A).^22^ The calibration plot showed minor deviations at lower and higher predicted probabilities (Fig. 2B; nevertheless, no systematic underestimation was observed in the high-probability range. These results suggest that the P-ROSC score may serve as a practical prognostic tool for prehospital risk stratification in Japan.

However, slight regional differences in predictive performance were observed. For example, the AUC was 0.772 in Hokkaido and 0.822 in Tohoku, and these regions also differed substantially in terms of prehospital adrenaline administration (36.3% vs. 19.7%). Because prehospital drug administration is one of the most heavily weighted variables in the P-ROSC score (Table S1), such variations in treatment may partly explain these differences. Additionally, regional heterogeneity in patient characteristics and prehospital circumstances—such as the proportion of arrests receiving bystander CPR and the aetiology of cardiac arrest (Table 1, Figs. S6 and S7)—may have further influenced the discriminative performance of the score. These observations align with the concept of spectrum bias, in which variations in case mix and treatment practices across subpopulations lead to small but measurable differences in predictive accuracy.^23^.

In addition to differences in predictive performance, the baseline incidence of ROSC also varied across regions. The proportion of patients achieving prehospital ROSC was higher in Hokkaido (10.7%) than in Tohoku (7.9%) and the overall cohort (9.5%) (Table 1). This regional variation may partly reflect differences in prehospital care intensity, including the higher rates of prehospital adrenaline administration and advanced airway management observed in Hokkaido. Physiologically, adrenaline may increase the likelihood of ROSC by enhancing coronary perfusion pressure through α-adrenergic vasoconstriction, while advanced airway management may support effective oxygenation and ventilation during resuscitation. Although these interventions have not consistently translated into improved long-term neurological outcomes, they may contribute to higher ROSC rates under specific clinical conditions.^24^, ^25^

Compared with external validations in other regions, the P-ROSC score performed well in Taiwan,^9^ where EMS arrival times were short and prehospital adrenaline administration was administered in 12.7% of cases, but was less effective in Thailand,^8^ where patients were generally younger, non-cardiac aetiologies were more common, and adrenaline use was nearly universal. These findings emphasise that the generalisability of the P-ROSC score in predicting prehospital ROSC depends on the characteristics of regional variations in patient characteristics and EMS systems.

Clinically, the P-ROSC score may help guide decisions regarding transport to advanced-care facilities. The use of higher cutoff values (e.g., ≥60 or ≥70) yielded a specificity greater than 90%, allowing the identification of patients with a high likelihood of achieving ROSC. At moderate cutoffs (e.g., ≥40 or ≥50), sensitivity and specificity were more balanced; however, decision-making at these thresholds should also consider contextual factors such as resuscitation duration, transport distance, and local medical resource availability. From the perspective of the termination of resuscitation (TOR), minimising false negatives is critical. At lower cutoff values (e.g., ≥10 or ≥20), sensitivity (92.8–98.5%) and NPV (98.4–98.9%) remained high, yet approximately 1.1% of patients with scores <10 still achieved ROSC. Previous studies have suggested a survival probability of ≤1% as a threshold for medical futility.^26^, ^27^, ^28^ Therefore, the P-ROSC score alone may be insufficient to support TOR decisions.

For future model refinement, it may be preferable to exclude or carefully interpret treatment-dependent variables such as drug administration. As noted by van Geloven et al.,^29^ the application of models to populations with treatment practices that differ from those of the development cohort can result in model miscalibration. When such treatment-dependent predictors are included, they may reflect institutional policies, intervention patterns, or system-level performance rather than patients’ intrinsic cardiac arrest characteristics. This raises important conceptual and ethical concerns about whether patients should receive worse predicted scores due to system performance and whether such scores may perpetuate outcome differences driven partly by healthcare systems rather than underlying pathology. Additionally, when prognostic scores are applied in clinical practice, caution is warranted if predictions are relied upon in isolation to inform critical decisions, such as the continuation or termination of resuscitative efforts. Overreliance on a single prognostic estimate may contribute to self-fulfilling prophecies, whereby predictions influence clinical decision-making and inadvertently reinforce the predicted outcomes.^30^ Accordingly, prediction scores should be interpreted within a broader, multimodal clinical assessment rather than used as standalone determinants of individual patient management.

This study has several limitations. First, the external validation was conducted exclusively within Japan. Further studies in other countries or regions are needed to confirm the generalisability of the score across diverse patient populations and EMS systems. Second, the application of a rigid, fixed-point prediction score across geographically diverse regions represents an inherent limitation, particularly in settings with substantial variation in EMS intensity and prehospital care practices. In this study, prehospital interventions and outcomes varied across regions, reflecting heterogeneity in EMS resources and treatment patterns. Accordingly, predicted risks should be interpreted in the context of local EMS characteristics, and the observed regional differences in predictive performance should be interpreted with caution because the study design does not allow causal attribution to specific factors. Third, the possibility of a self-fulfilling prophecy cannot be excluded. Prognostic estimates may influence the intensity or continuation of resuscitative efforts, potentially reinforcing predicted outcomes and leading to an apparent overestimation of model performance. However, because termination of resuscitation is not legally permitted in the prehospital setting in Japan (Supplementary Appendix 1), the magnitude of this bias may be limited. Finally, this study did not evaluate how the P-ROSC score would perform when implemented in real-world clinical workflows. Future research is needed to assess its impact on prehospital decision-making and downstream patient outcomes.

## Conclusion

In this nationwide external validation study, the P-ROSC score demonstrated fair discrimination and moderate calibration for predicting prehospital ROSC among patients with OHCA in Japan. Regional differences in predictive performance likely reflect variations in EMS systems and prehospital interventions. As the score incorporates adrenaline administration, which is a treatment-dependent variable, its application should be interpreted with caution, and further refinement may enhance its generalisability and clinical utility.

## Source of funding

This study was supported by the Japan Society for the Promotion of Science KAKENHI (grant numbers: JP23K15628 to NN and JP22H03313 to TI).

## CRediT authorship contribution statement

**Ryuta Onodera:** Writing – review & editing, Writing – original draft, Visualization, Software, Methodology, Formal analysis, Data curation, Conceptualization. **Norihiro Nishioka:** Writing – review & editing, Methodology, Funding acquisition, Data curation. **Yohei Okada:** Writing – review & editing, Data curation. **Yuto Makino:** Writing – review & editing, Data curation. **Kosuke Kiyohara:** Writing – review & editing, Formal analysis, Data curation. **Tetsuhisa Kitamura:** Project administration, Formal analysis, Data curation. **Taku Iwami:** Writing – review & editing, Supervision, Project administration, Funding acquisition, Conceptualization.

## Declaration of competing interest

The authors declare the following financial interests/personal relationships which may be considered as potential competing interests: YO received the research fund from the ZOLL Foundation. The authors declare no conflict of interest.

## Data Availability

Data supporting the results of this study are available from the corresponding author upon reasonable request.
