# Supplementary material for: External validation of the Prehospital Return of Spontaneous Circulation (P-ROSC) score for predicting prehospital return of spontaneous circulation among patients with out-of-hospital cardiac arrest
Source: Resusc Plus. 2026 Mar 19;29:101294. doi: 10.1016/j.resplu.2026.101294 (PMC13062545; doi:10.1016/j.resplu.2026.101294)
Supplement: Supplementary Data 1 [file mmc1.docx]

**Supplementary material**

**External validation of the prehospital return of spontaneous circulation**

**(P-ROSC) score for predicting prehospital return of spontaneous circulation among patients with out-of-hospital cardiac arrest**

**Contents**

| **Supplementary Appendix 1** | **Description of the All-Japan Utstein Registry** |
| --- | --- |
| **Table S1** | **TRIPOD checklist for reporting of the external validation of the P-ROSC score** |
| **Table S2** | **Score table of the P-ROSC score** |
| **Table S3** | **Definition of variables** |
| **Table S4** | **Missing data** |
| **Table S5** | **Comparison of baseline characteristics between development and validation cohorts** |
| **Table S6** | **Cutoff points and predictive performance of the P-ROSC score** |
| **Fig. S1** | **Regional classification of Japan** |
| **Fig. S2** | **Discriminative performance of the P-ROSC score by region: Area Under the receiver operating characteristic curve (AUC) with 95% confidence intervals (CIs)** |
| **Fig. S3** | **Calibration plots for P-ROSC score validation across different regions and the overall cohort** |
| **Fig. S4** | **Decision curve analysis of the P-ROSC score for rule-in decisions** |
| **Fig. S5** | **Receiver operating characteristic curves and calibration plots of the PROSC score according to sex** |
| **Fig. S6** | **Receiver operating characteristic curves and calibration plots of the PROSC score according to bystander CPR** |
| **Fig. S7** | **Receiver operating characteristic curves and calibration plots of the PROSC score according to aetiology** |

**Supplementary Appendix 1. Description of the All-Japan Utstein Registry**

The All-Japan Utstein Registry covers approximately 127 million people across a geographical area of about 378,000 km². Japan is geographically diverse, and the transport environments and outcomes of out-of-hospital cardiac arrest (OHCA) vary across regions.[1] The regions included in this study comprised the Kanto area, centred around Tokyo and characterised by a high population density and numerous large medical institutions; the Hokkaido and Tohoku areas, which are known for their wide transport distances and cold climates; the Chubu and Kinki areas, which encompass a mix of urban and rural settings; and the Chugoku, Shikoku, and Kyushu areas, which include regions with a high proportion of older adults.[2,3]

Cardiac arrest is defined as the complete cessation of mechanical cardiac activity, as confirmed by emergency medical service (EMS) personnel through the absence of signs of circulation.[4] According to the current Utstein-style template, cardiac arrest is presumed to be of medical origin unless attributed to trauma, drug overdose, drowning, electrocution, or asphyxia.[5] These diagnoses were made clinically by physicians working together with EMS personnel. All OHCA survivors were followed for 1 month after the event by the responsible EMS teams. EMS personnel completed data forms in collaboration with the attending physician. This information was then transferred and integrated into the registration system on the Fire and Disaster Management Agency (FDMA) database server. The data were subsequently verified at the terminals and validated by the FDMA. If incomplete data forms were identified, the responsible EMS personnel were requested to finalise them.

**EMS system in Japan**

In Japan, EMS is provided by regional governments and, as mentioned earlier, there were 750 fire departments with dispatch centres in 2015.[6] Emergency life-saving technicians (ELSTs) are expertly trained to provide critical care, including establishing intravenous lines, inserting adjunct airways, and using semi-automated external defibrillators in patients experiencing OHCA. With additional training, ELSTs can perform endotracheal intubation and administer adrenaline. Each ambulance typically includes three emergency providers, at least one of whom is an ELST. Management of cardiac arrest follows the Japanese cardiac pulmonary resuscitation (CPR) guidelines.[7] In Japan, EMS personnel are generally not legally permitted to terminate resuscitation in the prehospital setting; therefore, most OHCA patients treated by EMS are transported to hospital. Therefore, except for cases involving decapitation, severe burns, decomposition, rigor mortis, or evident dependent cyanosis, most patients with OHCA treated by EMS personnel are transported to hospitals, and relevant data are recorded in the All-Japan Utstein Registry.

Although the EMS system in Japan is nationally standardised under the Japanese CPR guidelines and legal frameworks, practical differences may exist across regions. For example, although the scope of practice for ELSTs—such as intubation and administration of adrenaline—is regulated and consistent nationwide, variations may arise in the actual implementation and quality of prehospital care. These include differences in the timeliness of emergency response, frequency of advanced airway use, adherence to CPR protocols, and experience level of EMS personnel.[1,8–10] Such variations may be influenced by factors such as urban–rural geography, EMS training resources, dispatch centre protocols, and the degree of public engagement with emergency services. Differences in the EMS system capacity and operational efficiency across prefectures may ultimately affect patient outcomes.[9]

**Supplementary Tables**

**Table S1.** TRIPOD checklist for reporting of the external validation of the P-ROSC score

| **Section/topic** | **Checklist item** | **Location in manuscript** |
| --- | --- | --- |
| **Title** | Identify the study as evaluating the performance of a multivariable prediction model, the target population, and the outcome | Title |
| **Abstract** | Structured summary of study design, participants, outcome, and performance measures | Abstract |
| **Introduction – Background** | Healthcare context and rationale for evaluating the prediction model | Introduction, paragraphs 1–3 |
|  | Description of target population and intended use of the model | Introduction, paragraphs 2–3 |
|  | Known health inequalities | Not applicable |
| **Objectives** | Specify that the study evaluates a prediction model | Introduction, final paragraph |
| **Data** | Data sources for model development and evaluation | Methods, Setting, Appendix 1 |
|  | Dates of data collection | Methods, Participants |
| **Participants** | Study setting, number and location of centres | Methods, Participants |
|  | Eligibility criteria | Methods, Participants |
|  | Treatments received and handling during evaluation | Methods, Appendix 1 |
| **Data preparation** | Data preprocessing and quality checks | Methods, Appendix 1 |
| **Outcome** | Definition of outcome and time horizon | Methods, Outcome, Table S3 |
|  | Outcome assessor characteristics | Methods, Appendix 1 |
|  | Blinding of outcome assessment | Not performed (objective outcome) |
| **Predictors** | Source and selection of predictors | Methods, Prediction model |
|  | Definition and measurement of predictors | Methods, Table S3 |
|  | Predictor assessor characteristics | Methods, Appendix 1 |
| **Sample size** | Explanation of sample size determination | Methods, Sample size |
| **Missing data** | Handling of missing data | Methods, Table S4 |
| **Statistical analysis** | Use of data for model evaluation | Methods, Statistical analysis |
|  | Handling of predictors in analyses | Methods, Statistical analysis |
|  | Model type and evaluation strategy | Methods, Statistical analysis |
|  | Assessment of heterogeneity across clusters | Methods, Subgroup analysis |
|  | Measures of model performance | Methods, Statistical analysis |
|  | Model updating or recalibration | Not performed |
|  | Calculation of model predictions | Methods, Statistical analysis |
| **Results – Participants** | Flow of participants | Figure 1 |
|  | Participant characteristics | Table 1 |
|  | Comparison with development data | Results, Table S5 |
| **Model performance** | Performance estimates with CIs | Results, Figure 2 |
|  | Heterogeneity of performance across clusters | Results, Fig. S2, S3, S5 |
| **Discussion – Interpretation** | Overall interpretation of results | Discussion |
| **Limitations** | Study limitations and generalisability | Discussion, Limitations |
| **Usability** | Handling unavailable or poor-quality input data | Discussion |
|  | Required expertise of users | Discussion |
|  | Future research directions | Discussion, final paragraph |
| **Ethics** | Ethics approval and consent | Methods, Study design |
| **Funding** | Funding source | Funding |
| **Conflicts of interest** | Declaration of interests | Declaration of competing interest |
| **Data sharing** | Provide details of data availability | Data availability statement |
| **Code sharing** | Provide details of analytical code availability | Data availability statement |

**Table S2.** Score table of the P-ROSC score

| **Variable** | **Score** |
| --- | --- |
| **Age (year)** | |
| <60 | 13 |
| 60–85 | 10 |
| 85–90 | 7 |
| ≥90 | 0 |
| **Time to EMS arrival (minutes)** | |
| <5 | 9 |
| 5–9 | 7 |
| 9–12 | 3 |
| ≥12 | 0 |
| **First rhythm** | |
| Non-shockable | 0 |
| Shockable | 30 |
| **Arrest witnessed** | |
| No | 0 |
| Professional | 27 |
| Lay person | 19 |
| **Prehospital drug administration** | |
| Yes | 21 |
| No | 0 |

P-ROSC, Prehospital Return of Spontaneous Circulation; EMS, emergency medical service.

**Table S3.** Definition of variables

| **Variables** | **Description** |
| --- | --- |
| Age | Age (years) |
| Sex | Sex (men/women) |
| Arrest witnessed | Witness of collapse (layperson/EMS personnel/not witnessed or unknown) |
| Cause of arrest | Cause of OHCA (cardiac/non-cardiac) |
| First rhythm | Initial cardiac rhythm at the scene, as confirmed by paramedics (shockable/non-shockable) |
| Prehospital drug administration | Adrenaline administration by EMS (yes/no) |
| Advanced airway by EMS | Advanced airway by EMS (yes/no) |
| Time to EMS arrival | Time duration between call to EMS and scene arrival (min) |
| Time to hospital arrival | Time duration between call to EMS and hospital arrival (min) |
| ROSC | ROSC at the scene or during transport, including both sustained and transient ROSC |
| Survival at 1 month | Survival status at 30 days after cardiac arrest (yes/no) |
| Favourable neurological survival at 1 month | Neurological status based on the Cerebral performance category (CPC) at 30 days after cardiac arrest (favourable/unfavourable)  Favourable: CPC, 1–2; Unfavourable: CPC, 3–5. |

EMS, emergency medical service; OHCA, out-of-hospital cardiac arrest; ROSC, return of spontaneous circulation.

**Table S4.** Missing data

| **Variables** | **Overall** |
| --- | --- |
| n | 493,695 |
| Age | 0 (0%) |
| Male | 0 (0%) |
| Witness | 0 (0%) |
| Cardiac cause | 0 (0%) |
| First documented rhythm | 0 (0%) |
| Adrenaline administration by EMS* | 0 (0%) |
| Advanced airway management by EMS* | 0 (0%) |
| Time to EMS arrival | 351 (0.07%) |
| Time to hospital arrival | 800 (0.16%) |
| ROSC | 0 (0%) |
| Survival at 1 month | 0 (0%) |
| Favourable neurological survival at 1 month | 0 (0%) |

*Prehospital therapeutic interventions are recorded only when they are performed; therefore, the absence of a record indicates that the intervention was not performed.

Values are expressed as numbers and percentages (%).

EMS, emergency medical services; ROSC, return of spontaneous circulation

**Table S5.** Comparison of baseline characteristics between development and validation cohorts

| **Variable** | **Validation (n = 493,695)** | **Development (n = 119,477)** |
| --- | --- | --- |
| **Age, years** | 81 [70–88] | 76 [62–84] |
| **Arrest witnessed** |  |  |
| Lay person | 170,300 (34.5) | 42,703 (35.7) |
| No | 292,653 (59.3) | 68,264 (57.1) |
| Professional | 30,742 (6.2) | 8,510 (7.1) |
| **Initial rhythm** |  |  |
| Shockable | 42,174 (8.5) | 11,096 (9.3) |
| Unshockable | 451,521 (91.5) | 108,381 (90.7) |
| **Prehospital drug administration** | 127,875 (25.9) | 14,649 (12.3) |
| **Time to EMS arrival, minutes** | 8.0 [6.0–10.0] | 6.0 [5.0–8.0] |
| **ROSC** | 46,760 (9.5) | 9,628 (8.1) |

Categorical variables are presented as n (%), and continuous variables as median [interquartile range].

EMS, emergency medical services; ROSC, return of spontaneous circulation

**Table S6.** Cutoff points and predictive performance of the P-ROSC score

| P-ROSC score  cut off (≥) | ROSC/Total, n (%) | Sensitivity  (95% CI), % | Specificity  (95% CI), % | PPV  (95% CI), % | NPV  (95% CI), % |
| --- | --- | --- | --- | --- | --- |
| 10 | 45,987/427,511 (10.8) | 98.5 (98.4–98.6) | 14.6 (14.5–14.7) | 10.8 (10.7–10.9) | 98.9 (98.8–99.0) |
| 20 | 43,329/281,377 (15.4) | 92.8 (92.5–93.0) | 46.7 (46.6–46.8) | 15.4 (15.3–15.5) | 98.4 (98.4–98.5) |
| 30 | 38,659/210,656 (18.4) | 82.8 (82.4–83.1) | 61.5 (61.3–61.6) | 18.4 (18.2–18.5) | 97.2 (97.1–97.2) |
| 40 | 28,116/112,206 (25.1) | 60.2 (59.8–60.6) | 81.2 (81.1–81.3) | 25.1 (24.8–25.3) | 95.1 (95.1–95.2) |
| 50 | 21,708/75,736 (28.7) | 46.5 (46.0–46.9) | 87.9 (87.8–88.0) | 28.7 (28.3–29.0) | 94.0 (93.9–94.1) |
| 60 | 11,908/34,506 (34.5) | 25.5 (25.1–25.9) | 94.9 (94.9–95.0) | 34.5 (34.0–35.0) | 92.4 (92.3–92.5) |
| 70 | 3,757/12,572 (29.9) | 8.0 (7.8–8.3) | 98.0 (98.0–98.1) | 29.9 (29.1–30.7) | 91.1 (91.0–91.1) |
| 80 | 1,964/8,059 (24.4) | 4.2 (4.0–4.4) | 98.6 (98.6–98.7) | 24.4 (23.4–25.3) | 90.8 (90.7–90.9) |
| 90 | 380/1,780 (21.4) | 0.8 (0.7–0.9) | 99.7 (99.7–99.7) | 21.3 (19.5–23.3) | 90.6 (90.5–90.7) |

CI, confidence interval; PPV, positive predictive value; NPV, negative predictive value; P-ROSC, Prehospital Return of Spontaneous Circulation.

**Supplementary Figures**

**
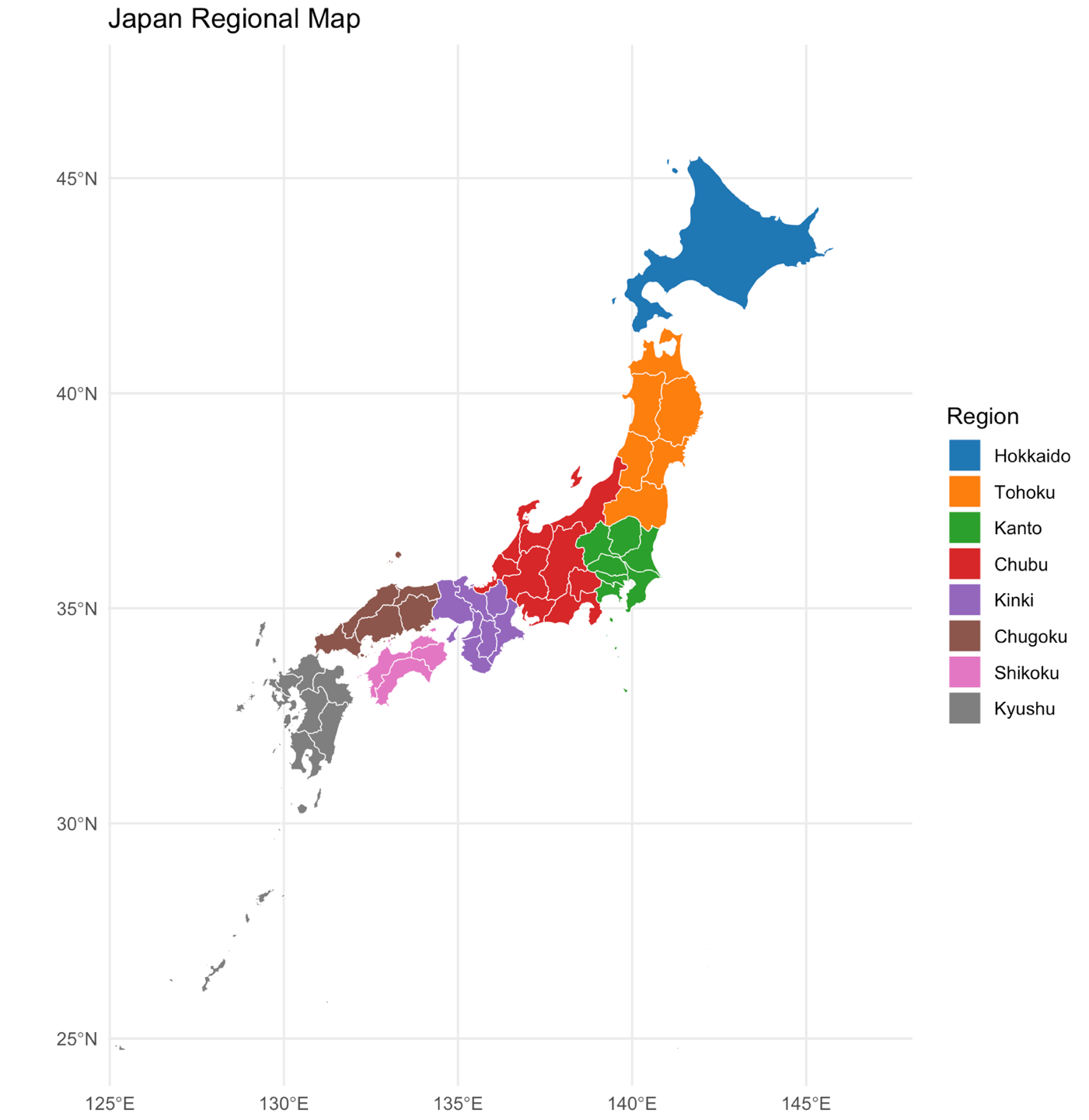
**

**Fig. S1.** Regional classification of Japan

This figure illustrates the regional classifications of Japan used in this study. The prediction accuracy of the Prehospital Return of Spontaneous Circulation score was evaluated separately for each region. This classification followed the standard regional divisions of Japan: Hokkaido (blue), Tohoku (orange), Kanto (green), Chubu (red), Kinki (purple), Chugoku (brown), Shikoku (pink), and Kyushu (gray).


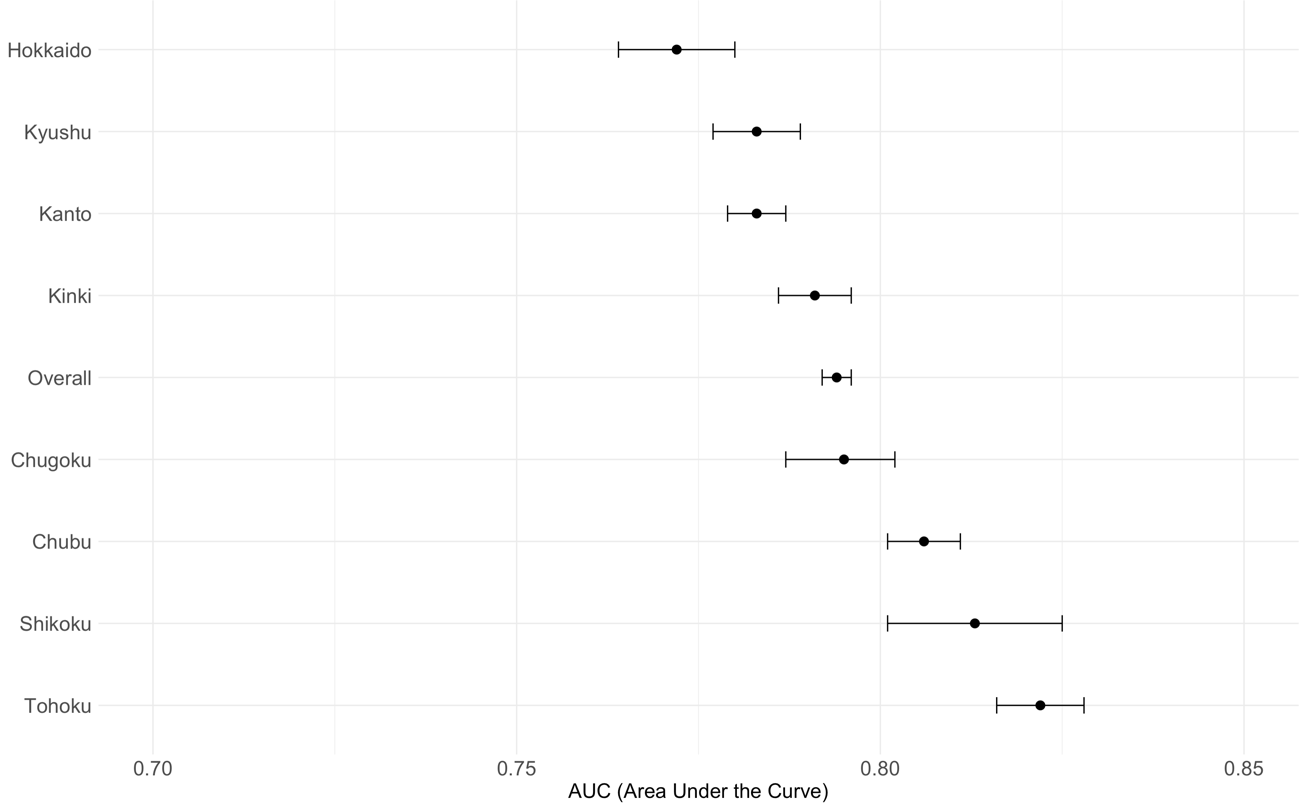


**Fig. S2.** Discriminative performance of the P-ROSC score by region: Area Under the receiver operating characteristic curve (AUC) with 95% confidence intervals (CIs).

The AUC, along with its 95% CIs, is shown for each geographic region in Japan and for the overall cohort. AUC reflects the discrimination performance of the P-ROSC score. Each dot represents the point estimate of the AUC, and horizontal lines indicate the corresponding 95% CIs. The regions are listed in ascending order of AUC values.


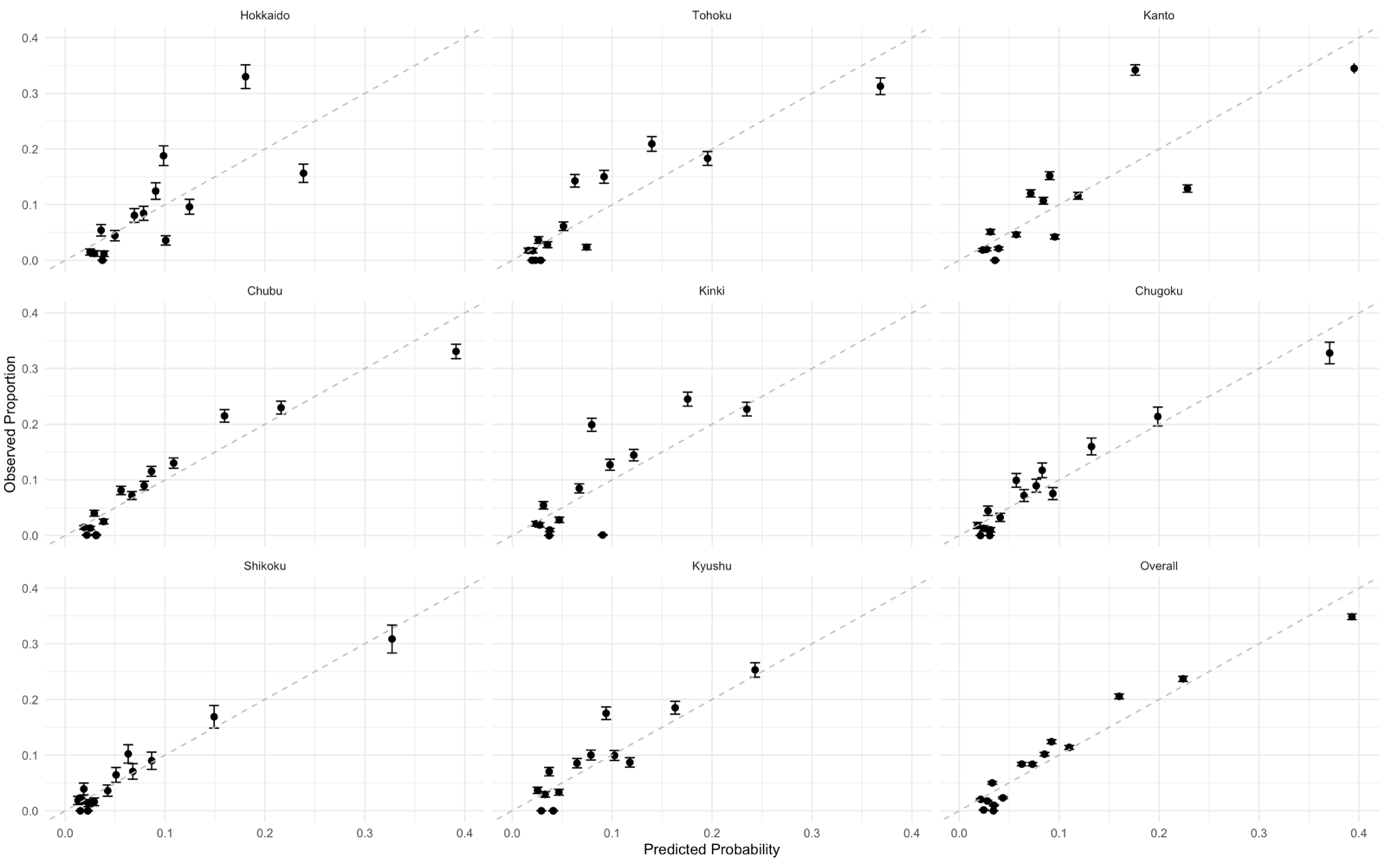


**Fig. S3.** Calibration plots for P-ROSC score validation across different regions and the overall cohort.

Calibration plots for external validation of the P-ROSC score across the eight regions and the overall cohort. The plots show the relationship between the predicted probabilities of prehospital ROSC and the observed proportions. Each point represents a decile of the predicted probability, with vertical bars indicating 95% confidence intervals. The dashed diagonal line represents a perfect calibration (ideal agreement between the predicted and observed values).

**
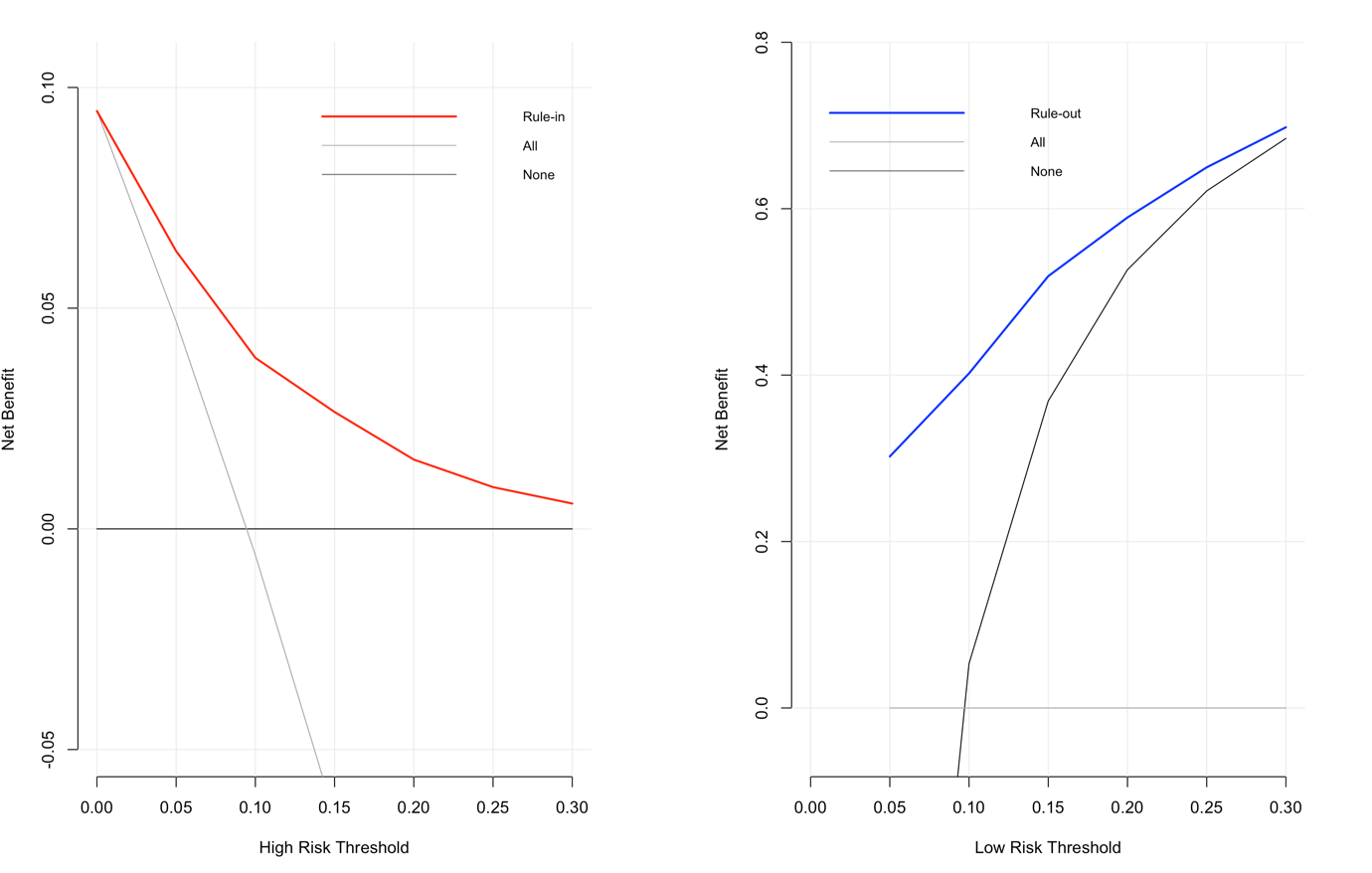
**

**Fig. S4.** Decision curve analysis of the P-ROSC score under rule-in and rule-out strategies

Model indicates the P-ROSC score–based strategy; All, treat-all strategy; None, treat-none strategy.

**
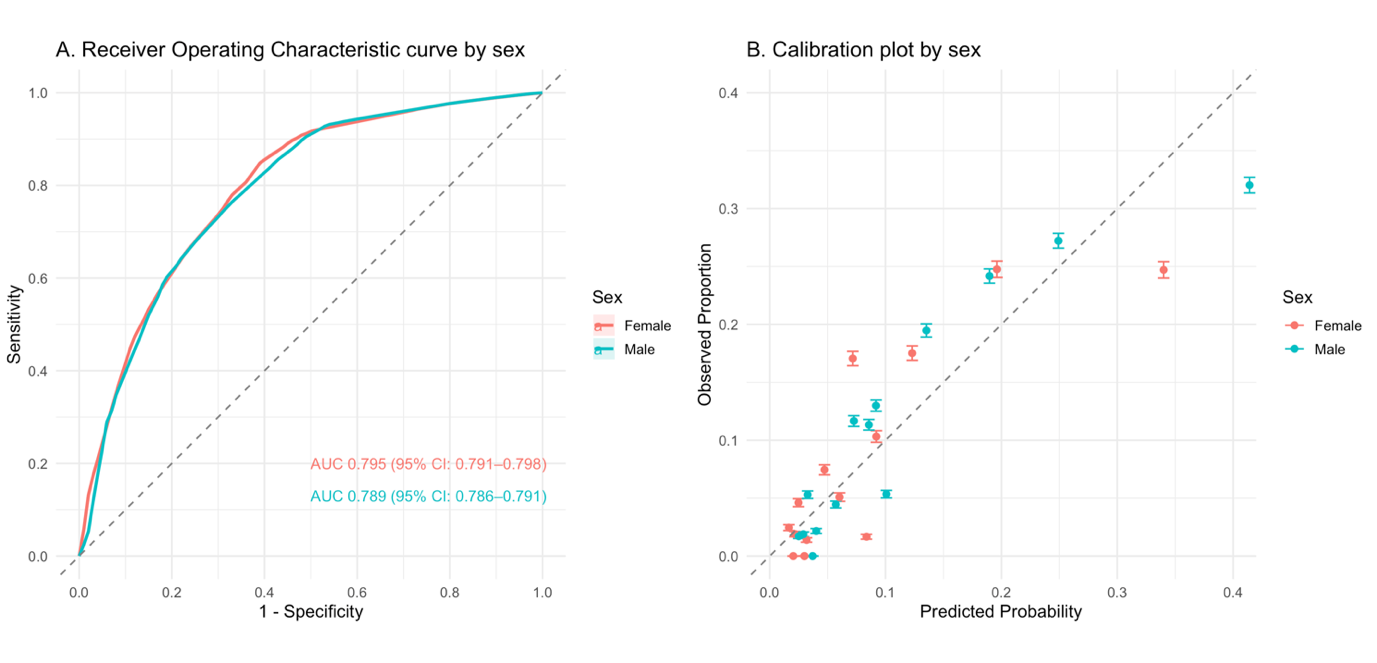
**

**Fig. S5.** Receiver operating characteristic curves and calibration plots of the PROSC score according to sex

**
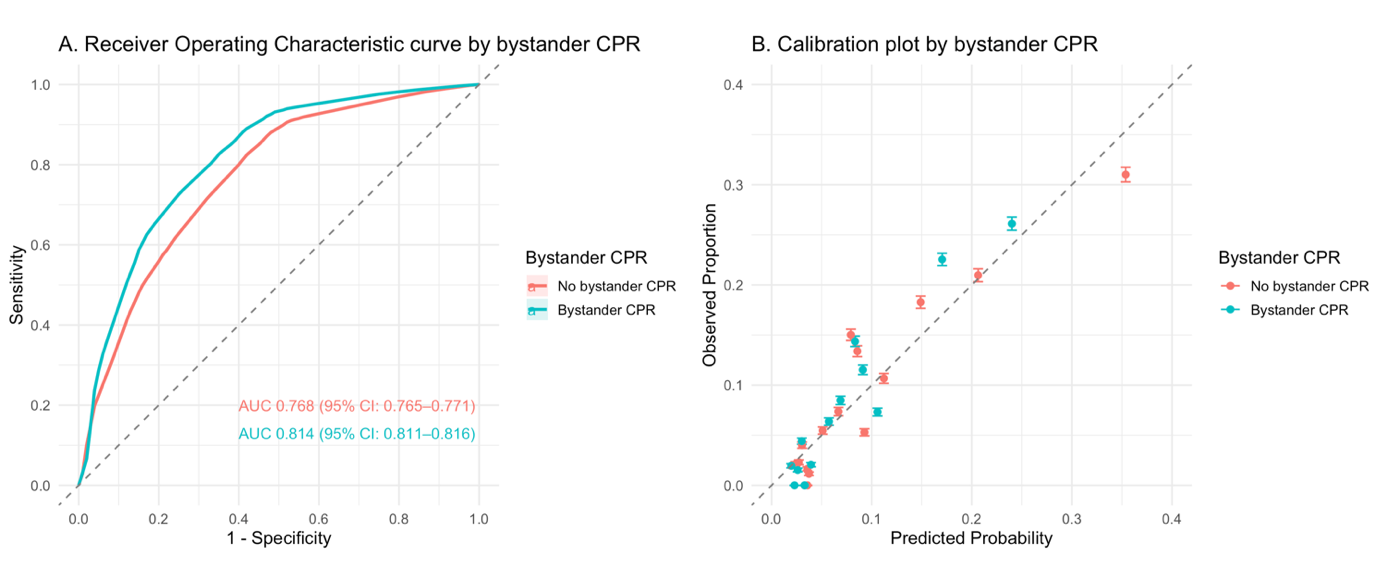
**

**Fig. S6.** Receiver operating characteristic curves and calibration plots of the PROSC score according to bystander CPR

CPR, cardiopulmonary resuscitation.

**
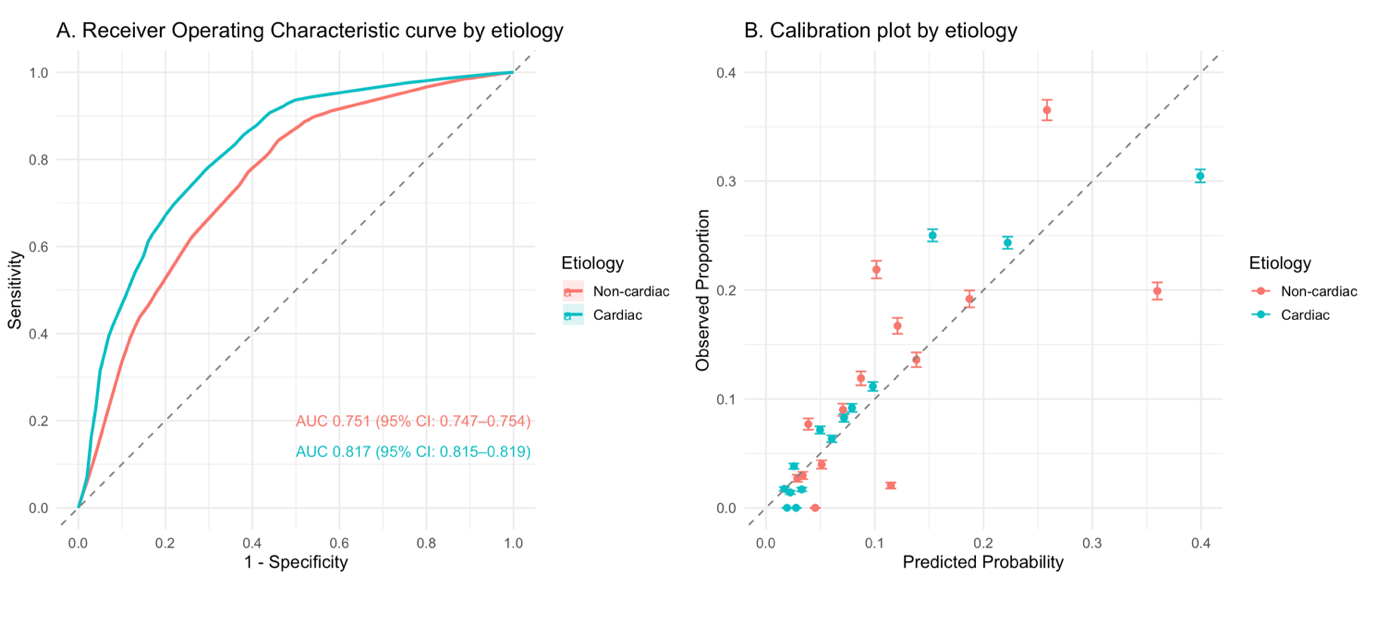
**

**Fig. S7.** Receiver operating characteristic curves and calibration plots of the PROSC score according to aetiology

**Reference**

[1] Okubo M, Gibo K, Wallace DJ, Komukai S, Izawa J, Kiyohara K, et al. Regional variation in functional outcome after out-of-hospital cardiac arrest across 47 prefectures in Japan. Resuscitation 2018;124:21–8. https://doi.org/10.1016/j.resuscitation.2017.12.030.

[2] Ministry of Health. Labour and Welfare. Survey of medical institutions 2023. https://www.mhlw.go.jp/toukei/list/79-1.html (accessed 21 May, 2025).

[3] Ministry of Internal Affairs and Communications. Population estimates based on Basic Resident Register 2023. https://www.soumu.go.jp/menu_news/s-news/01gyosei02_02000316.html (accessed 21 May, 2025).

[4] Kitamura T, Iwami T, Kawamura T, Nitta M, Nagao K, Nonogi H, et al. Nationwide improvements in survival from out-of-hospital cardiac arrest in Japan. Circulation 2012;126:2834–43. https://doi.org/10.1161/CIRCULATIONAHA.112.109496.

[5] Bray JE, Grasner J-T, Nolan JP, Iwami T, Ong MEH, Finn J, et al. Cardiac Arrest and Cardiopulmonary Resuscitation Outcome Reports: 2024 Update of the Utstein Out-of-Hospital Cardiac Arrest Registry Template. Circulation 2024;150:e203–23. https://doi.org/10.1161/CIR.0000000000001243.

[6] Nishiyama C, Kiyohara K, Matsuyama T, Kitamura T, Kiguchi T, Kobayashi D, et al. Characteristics and outcomes of out-of-hospital cardiac arrest in educational institutions in Japan ― All-Japan Utstein registry ―. Circulation Journal 2020;84:1234-42. https://doi.org/10.1253/circj.CJ-19-0920.

[7] Japan Resuscitation Council. 2015 Japanese guidelines for emergency care and cardiopulmonary resuscitation (in Japanese). Tokyo: Igaku-Shoin 2016.

[8] Izawa J, Komukai S, Gibo K, Okubo M, Kiyohara K, Nishiyama C, et al. Pre-hospital advanced airway management for adults with out-of-hospital cardiac arrest: nationwide cohort study. BMJ 2019:364:l430. https://doi.org/10.1136/bmj.l430.

[9] Kawai Y, Yamamoto K, Miyazaki K, Asai H, Fukushima H. Machine learning-based analysis of regional differences in out-of-hospital cardiopulmonary arrest outcomes and resuscitation interventions in Japan. Sci Rep 2023;13:43210 https://doi.org/10.1038/s41598-023-43210-x.

[10] Okamoto Y, Iwami T, Kitamura T, Nitta M, Hiraide A, Morishima T, et al. Regional variation in survival following pediatric out-of-hospital cardiac arrest. Circulation Journal 2013;77:1772-8. https://doi.org/10.1253/circj.CJ-12-1604.
